# Measurements of the Relationship Between Microstructure, pH, and the Streaming and Zeta Potentials of Sandstones

**DOI:** 10.1007/s11242-017-0954-5

**Published:** 2017-11-13

**Authors:** E. Walker, P. W. J. Glover

**Affiliations:** 1 Département de géologie et de génie géologique, Université Laval, Quebec, G1V 0A6 Canada; 2 School of Earth and Environment, University of Leeds, Leeds, LS2 9JT UK

**Keywords:** Microstructure, Porosity, Streaming potential, Zeta potential, pH, Electrokinetic rock properties

## Abstract

A large number (1253) of high-quality streaming potential coefficient ($$C_\mathrm{sp})$$ measurements have been carried out on Berea, Boise, Fontainebleau, and Lochaline sandstones (the latter two including both detrital and authigenic overgrowth forms), as a function of pore fluid salinity ($$C_\mathrm{f})$$ and rock microstructure. All samples were saturated with fully equilibrated aqueous solutions of NaCl (10$$^{-5}$$ and 4.5 mol/dm$$^{3})$$ upon which accurate measurements of their electrical conductivity and pH were taken. These $$C_\mathrm{sp}$$ measurements represent about a fivefold increase in streaming potential data available in the literature, are consistent with the pre-existing 266 measurements, and have lower experimental uncertainties. The $$C_\mathrm{sp}$$ measurements follow a pH-sensitive power law behaviour with respect to $$C_\mathrm{f}$$ at medium salinities ($$C_\mathrm{sp} =-\,1.44\times 10^{-9} C_\mathrm{f}^{-\,1.127} $$, units: V/Pa and mol/dm$$^{3})$$ and show the effect of rock microstructure on the low salinity $$C_\mathrm{sp}$$ clearly, producing a smaller decrease in $$C_\mathrm{sp}$$ per decade reduction in $$C_\mathrm{f}$$ for samples with (i) lower porosity, (ii) larger cementation exponents, (iii) smaller grain sizes (and hence pore and pore throat sizes), and (iv) larger surface conduction. The $$C_\mathrm{sp}$$ measurements include 313 made at $$C_\mathrm{f} > 1$$ mol/dm$$^{3}$$, which confirm the limiting high salinity $$C_\mathrm{sp}$$ behaviour noted by Vinogradov et al., which has been ascribed to the attainment of maximum charge density in the electrical double layer occurring when the Debye length approximates to the size of the hydrated metal ion. The zeta potential ($$\zeta $$) was calculated from each $$C_\mathrm{sp}$$ measurement. It was found that $$\zeta $$ is highly sensitive to pH but not sensitive to rock microstructure. It exhibits a pH-dependent logarithmic behaviour with respect to $$C_\mathrm{f}$$ at low to medium salinities ($$\zeta =0.01133 \log _{10} \left( {C_\mathrm{f} } \right) +0.003505$$, units: V and mol/dm$$^{3})$$ and a limiting zeta potential (zeta potential offset) at high salinities of $${\zeta }_\mathrm{o} = -\,17.36\pm 5.11$$ mV in the pH range 6–8, which is also pH dependent. The sensitivity of both $$C_\mathrm{sp}$$ and $$\zeta $$ to pH and of $$C_\mathrm{sp}$$ to rock microstructure indicates that $$C_\mathrm{sp}$$ and $$\zeta $$ measurements can only be interpreted together with accurate and equilibrated measurements of pore fluid conductivity and pH and supporting microstructural and surface conduction measurements for each sample.

**Electronic supplementary material:**

The online version of this article (10.1007/s11242-017-0954-5) contains supplementary material, which is available to authorized users.

## Introduction

**Fig. 1:**
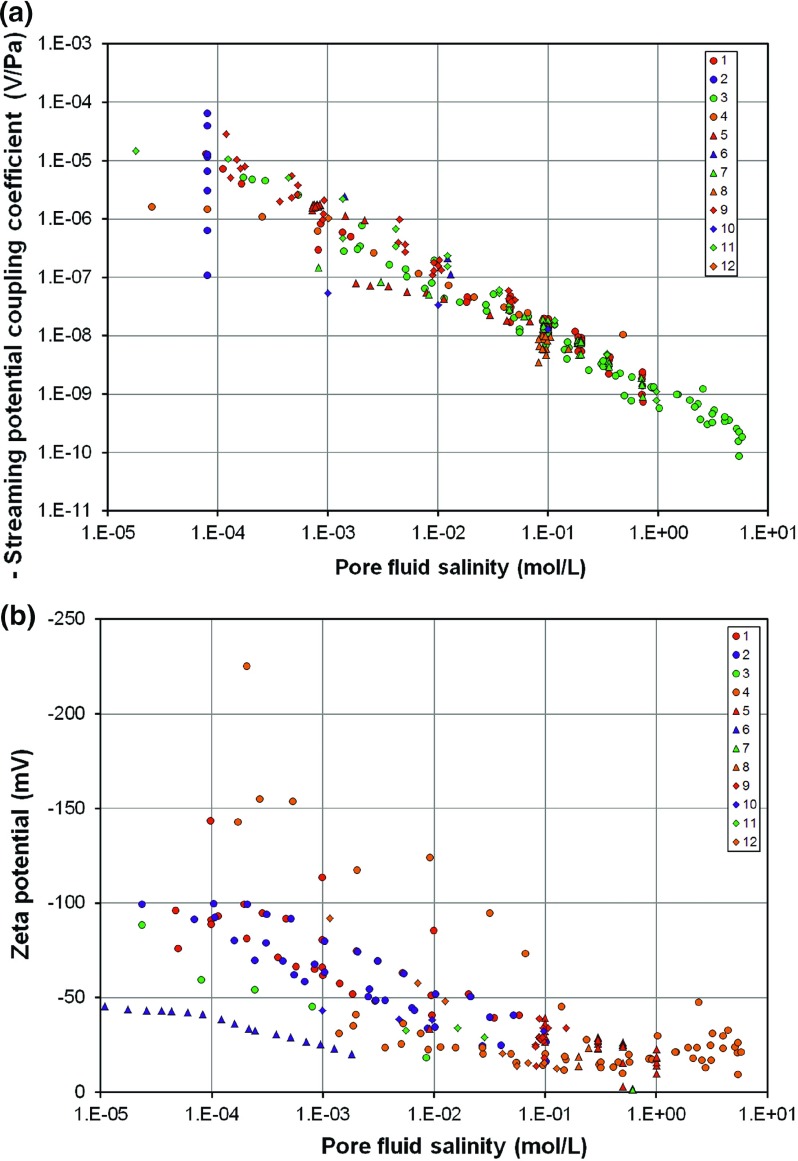
**a** A compilation of 266 experimentally measured values of the streaming potential coefficient as a function of pore fluid salinity. **b** A compilation of 269 experimentally measured values of the zeta potential coefficient as a function of pore fluid salinity. All values are negative in both parts of the figure. Temperature 20–$25^{\,\circ }\hbox {C}$. pH5–9 for all measurements. **a** [1] sandstone with NaCl (Sprunt et al. 1994; Jouniaux and Pozzi 1995a, 1997; Li et al. 1995; Jiang et al. 1998; Pengra et al. 1999); [2] sandstone with NaCl as a function of permeability/microstructure (pH5) (Jouniaux and Pozzi 1995b); [3] St. Bees, Stainton, and Fontainebleau sandstones with NaCl (Jaafar et al. 2009; Vinogradov et al. 2010); [4] sandstone with KCl (Alkafeef and Alajmi 2007); [5] sand with NaCl (Guichet et al. 2003; Block and Harris 2006); [6] granite with NaCl (Morgan et al. (1989)); [7] glass with NaCl (Pengra et al. 1999; Block and Harris 2006); [8] zeolitized tuffs with NaCl (Revil et al. 2002); [9] basalt with NaCl (Revil et al. 2003); [10] granite with KCl (Tosha et al. 2003); [11] silica nanochannel with KCl (Heyden et al. 2006); [12] Ottawa sand (Tardif et al. 2011; Glover et al. 2012a). **b** [1] Quartz with NaCl (Pride and Morgan (1991)); [2] silica with NaCl (Gaudin and Fuerstenau 1955; Li and Bruyn 1966; Kirby and Hasselbrink 2004); [3] glass beads with NaCl (Bolève et al. 2007); [4] St. Bees, Stainton, and Fontainebleau sandstones with NaCl (Jaafar et al. 2009; Vinogradov et al. 2010); [5] clay minerals with NaCl (Kosmulski and Dahlsten 2006; Avena and Pauli 1998); [6] sandstone with KCl (Lorne et al. 1999); [7] quartz with NaCl (Kosmulski et al. 2002); [8] kaolin-coated sandstone with NaCl (Pengra et al. 1999); [9] tuff samples containing clays and zeolites (Revil et al. 2002); [10] kaolinite with NaCl (Poirier and Cases 1985); [11] mica with NaCl (Will and Nover 1986); [12] sandstone with NaCl (Alkafeef and Alajmi 2007)

The last 15 years have seen the publication of a number of laboratory studies of the streaming potential coefficient ($C_\mathrm{sp})$ and zeta potential ($\zeta$) of rocks, predominantly silica-rich mineralogies (e.g. Jouniaux and Pozzi 1995a, b, 1997; Alkafeef and Alajmi 2007; Vinogradov et al. 2010). Glover et al. (2006) collated a database of $C_\mathrm{sp}$ and $\zeta$ determinations as a function of salinity, which is reproduced in Fig. 1 with supplementary data from AC measurements on Ottawa sand (Tardif et al. 2011; Glover et al. 2012a), and omitting carbonate samples. Four aspects of the data are worth mentioning explicitly.

First, at low salinities there is a spread of $C_\mathrm{sp}$ values encompassing up to three orders of magnitude. Some of this spread is almost certainly due to experimental uncertainties, perhaps with the measurement itself, but most of the scatter is likely to be associated with the lack of pore fluid equilibration or knowledge of the real electrical conductivity, salinity, and pH of the fluid in the sample at the time of the measurement (Walker et al. 2014).

Second, the spread of $C_\mathrm{sp}$ at low salinities is also due to the effect of the rock microstructure (Jouniaux and Pozzi 1995b; Glover and Déry 2010; Glover et al. 2012b).

Third, the behaviour of both $C_\mathrm{sp}$ and $\zeta$ changes depending on the salinity. Attempts have been made previously to characterize the medium and high salinity behaviours of both $C_\mathrm{sp}$ and $\zeta$, the former as a power law with respect to salinity (e.g. Vinogradov et al. 2010), and the latter as a logarithmic relationship with respect to salinity in the medium salinity range (e.g. Pride and Morgan 1991; Bolève et al. 2007; Revil et al. 1999; Jaafar et al. 2009; Vinogradov et al. 2010) while taking a constant value in the high salinity range (e.g. Johnson et al. 1999a, b; Dukhin et al. 2005; Jaafar et al. 2009; Vinogradov et al. 2010). Higher-quality measurements are needed to confirm and improve such relationships, as well as allowing their dependence on other factors such as pH to be studied.

Fourth, it is extremely difficult to make measurements of the $C_{\mathrm{sp}}$ with high salinity fluids. Highly saline fluids are difficult to handle in a stable fashion, precipitating with small changes in temperature (Vinogradov et al. 2010). Furthermore, the values of $C_{\mathrm{sp}}$ are so low (Fig. 1a) that it is difficult to make accurate measurements of the resulting small streaming potentials.

There are a number of questions which arise from Fig. 1 and the points made above. These are: (i) to what extent in the scatter observed in the $C_{\mathrm{sp}}$ and $\zeta$ data caused by uncertainties in measurement and lack of control of parameters upon which $C_{\mathrm{sp}}$ and $\zeta$ depend? (ii) What are the main controlling factors on $C_{\mathrm{sp}}$ and $\zeta$, and can relationships between $C_{\mathrm{sp}}$, $\zeta$, and salinity be defined accurately? (iii) How does pH affect $C_{\mathrm{sp}}$ and $\zeta$ and their relationship with salinity? (iv) How does the microstructure of the samples affect $C_{\mathrm{sp}}$ and $\zeta$ ? (v) Can the high salinity behaviour of $C_{\mathrm{sp}}$ and $\zeta$ observed by Jaafar et al. (2009) and Vinogradov et al. (2010) be confirmed and quantified?

Consequently, the primary aim of the work described in this paper was to make the best possible measurements of $C_{\mathrm{sp}}$ and determinations of $\zeta$ as a function of salinity on sandstones over the largest range of salinities and especially including salinities greater than 1 mol/dm$^{3}$. These high-quality measurements would then allow us answer the questions posed above as well as to provide high-quality experimental data against which the theoretical model of Glover and Déry (2010) and Glover et al. (2012b) could be rigorously tested. This paper addresses the questions posed above and reserves the modelling to a forthcoming paper.

## Sample Material

Fourteen samples of four sandstones were measured, three samples of each of Berea and Boise sandstone, and four samples of each of Lochaline; Fontainebleau sandstones. Both Fontainebleau and Lochaline sandstones have two grades, one composed of subrounded detrital grains, the other consisting of detrital grains with additional euhedral quartz overgrowths. Our samples consisted of two of each type and can be distinguished by the additional letter ‘D’ for the detrital type and ‘Q’ for quartz overgrown type in the sample codes.

**Fig. 2:**
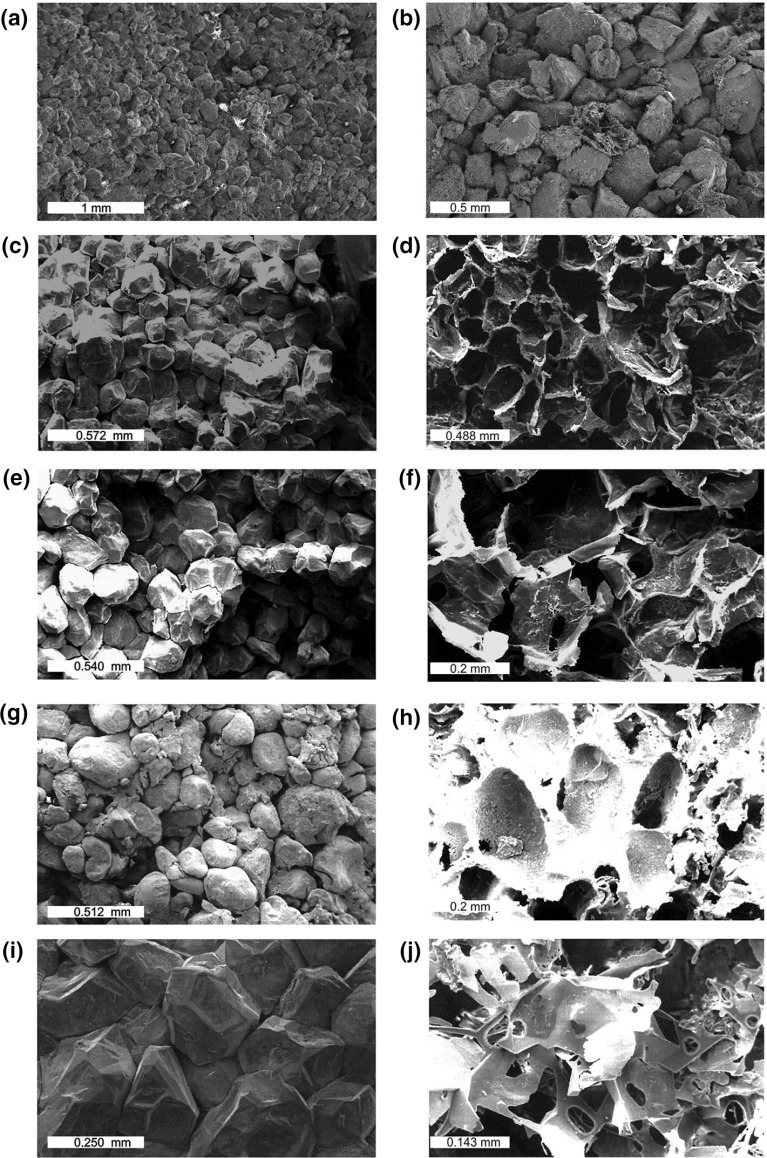
Photo-micrograph SEM images of **a** Berea sandstone (BR1), **b** Boise sandstone (B3I), **c** Fontainebleau sandstone (detrital, F1D) together with **d** its resin cast (F1D), **e** Fontainebleau sandstone (quartz overgrown, F4Q) together with **f** its resin cast (F4Q), **g** Lochaline sandstone (detrital, L2D) together with **h** its resin cast (L2D), **i** Lochaline sandstone (quartz overgrown, L3Q) together with **j** its resin cast (L3Q)

Each sample was submitted to a battery of basic measurements. The grain size distribution of crushed offcuts of each sample was measured by laser diffractometry using a Malvern Mastersizer 2000. The theta transformation (Glover and Walker 2009) was then used to calculate the pore size distribution of the rock and hence calculate the pore throat diameter with the method of Glover and Déry (2010). This approach is more accurate than using X-ray computed microtomography data. The pore throat diameter distribution was measured independently by mercury injection. Porosity measurements were taken using at least three techniques for most samples. These core porosity measurements are helium pycnometry, mercury injection porosimetry, and fluid saturation/Archimedes’ porosity. Some samples were also subjected to image analysis, X-ray computed tomography (CT), X-ray computed microtomography ($\upmu$CT), and NMR-based porosity measurements. A simple klinkenberg nitrogen permeability measurement was also taken on each sample. Fluid permeametry and impedance spectroscopy were also carried out during the main experimental runs. Data for all the samples are summarized in Table 1, while SEM photomicrographs of each rock are shown in Fig. 2.

We have carried out the procedure of making resin casts of the pore networks of both types of Fontainebleau and Lochaline sandstones. Initially, the samples were injected with low-viscosity epoxy resin by submerging them in resin in a vacuum oven heated to $50^{\,\circ }\hbox {C}$. The viscosity of the resin is sufficiently low at $50^{\,\circ }\hbox {C}$ to allow vacuum impregnation into pores as small as 1 $\upmu$m. In this work we also added a small confining pressure of 500 psi (3.45 MPa) using an old heated Hassler cell in order to fill the smallest spaces possible. Even then, we found that it was difficult to impregnate the thinnest intergranular pore sheets between the euhedral grains of the overgrown sandstones, the evidence of which can be found in the subcircular windows in the centre of some of the resin sheets (see Fig. 2j). These windows represent pore space too thin for the resin to access. The samples were then cut into small 10-mm cubes, placed in plastic containers, and treated with dilute hydrochloric acid to remove any carbonates. The latter step is required because insoluble fluorides may be formed if carbonates are present when the samples are placed in hydrofluoric acid. Finally, the samples were thoroughly cleaned and then submerged in warm $(50^{\,\circ }\hbox {C})$ hydrofluoric acid for several weeks in order to remove all traces of the silicate minerals. In practice, we found that there always remained a small core of undigested silicates at the centre of the sample, but this did not impede the imaging of the surface structures. Finally, the samples were thoroughly cleaned and then prepared for the SEM in the usual way.

The resulting ‘negative’ of the rock shows how the distribution of the pore space differs between the uncemented and cemented forms of the rock. Figure 2d, g, h, j shows SEM images of the resulting pore networks. In the detrital forms of both the Fontainebleau and Lochaline sandstones, the grain casts are subrounded and there is a significant volume of epoxy between the grain casts, indicating that the pore space is well-developed and interconnected, especially for the Lochaline sandstone (Fig. 2h). The saturation of this relatively large interdetrital porosity with conductive fluids results in high permeabilities and electrical conductivities, as well as cementation exponents that approach the values expected for spherical grains ($m = 1.5$), as shown in Table 1, and explaining the low mechanical strength.

**Table 1:** Sample properties

Parameter	Symbol	Units	Berea sandstone			Boise sandstone			Fontainebleau sandstone				Lochaline sandstone			
Sample codes			BR1 ET5	BR2	BR3	B1II	B2II	B3I	F1D	F2D	F3Q	F4Q	L1D O6	L2D O2	L3Q H3	L4Q H1
Modal grain diameter (laser diffractometry)	$d_\mathrm{grain}$	$\upmu$m	180	155	162	739	991	2140	235	231	251	255	263	268	277	278
Modal pore throat diameter (MICP)	$d_\mathrm{pore}$	$\upmu$m	8.35	6.27	14.24	34.8	34.8	74	13.2	14.8	0.46	0.68	15.5	18.2	1.19	2.58
Modal pore diameter [calculated, Glover and Walker (2009)]	$d_\mathrm{pore}$	$\upmu$m	17.04	14.68	16.14	125	181	274	20.4	22.4	0.48	0.54	23.2	24.1	0.99	1.99
Modal pore throat diameter [calculated, Glover and Déry (2010)]	$d_\mathrm{pt}$	$\upmu$m	10.23	8.82	9.69	75.3	109	165	12.3	13.5	0.29	0.32	13.95	14.53	0.60	1.20
Cementation exponent	*m*	(–)	1.79	1.72	1.66	1.569	1.677	1.811	1.71	1.68	1.60	1.63	1.5	1.55	1.54	1.48
Formation factor using *m* and ${\phi }_\mathrm{sat}$	*F*	(–)	10.22	10.63	10.47	6.53	5.65	7.48	11.6	10.6	567	503	13.1	12.4	312	163
Helium porosity	${\phi }_\mathrm{He}$	(–)	0.274	0.258	0.252	0.370	0.342	0.333	0.241	0.249	0.020	0.026	0.187	0.208	0.027	0.035
Saturation porosity	${\phi }_\mathrm{sat}$	(–)	0.272	0.253	0.243	0.302	0.356	0.329	0.238	0.245	0.019	0.022	0.180	0.197	0.024	0.032
Mercury porosity	${\phi }_\mathrm{Hg}$	(–)	0.254	0.232	0.228	0.300	0.259	0.257	0.235	0.235	0.015	0.018	0.210	0.205	0.022	0.030
Surface conductivity	${\Sigma }_{s}$	$\times ~10^{-4}$ S/m	285	66.5	50.2	13.6	32	13.4	2.44	2.31	62.2	55.4	1.96	1.81	21.85	10.1
pH	*pH*	(–)	8.03	7.05	7.41	7.2	5.94	6.61	6.40	6.41	6.48	6.51	7.27	7.26	7.15	7.12
Number of measurements	*N*	(–)	55	143	135	74	18	18	113	99	104	95	100	103	99	97

Note: Further information concerning the general properties of Boise, Berea, Fontainebleau and Loachaline sandstones may be found in Churcher et al. (1991); Gomez et al. (2010), Pasqualini et al. (2007), and Worden and Morad (2009)

By contrast the overgrown samples of both Fontainebleau and Lochaline sandstones show interlocking euhedral grains, which manifest themselves as thin sheets of porosity where two crystal faces grow together. Figure 2g, j shows these fine planar pore spaces, some of which are small enough not to allow the ingress of epoxy. These thin but highly connected pore spaces result in porosities ten times lower than in the detrital rocks (Table 1) with concomitantly smaller permeabilities. The slightly lower cementation exponents in these rocks are associated with the increased connectivity of the pore space, while interlocking grains ensure that the rock has a relatively high mechanical strength compared to the detrital form.

## Apparatus and Methodology

The experimental cell and measurement set-up is described in Walker et al. (2014). The pore fluids were made up to approximately the correct salinity using distilled, deionized water that had been previously degassed. The electrical conductivity and pH of the initial solution and that measured after equilibration throughout the measurements were measured using bench-top fluid conductivity and pH meters (VWR SB70C and SB70P, respectively), and the salinity of the solution was derived using the method of Sen and Goode (1992a, b) as a function of temperature. Once the samples were loaded and full saturation had been attained, the process fluid was circulated through the rock in a closed system until it had attained complete equilibrium with the sample. This was carried out while measuring the electrical conductivity and pH of fluids leaving the sample using both an in-line technique and by testing aliquots of the fluid. Initially we allowed 50 pore volumes (about 2000 cm$$^{3})$$ and the entire recycling of the input reservoir twice (8000 cm$$^{3})$$, whichever was greater, for this to occur. At the highest pump flow rate this would take about 16 h to complete. However, the measurements on the fluids leaving the cell were a useful monitor of when equilibration was attained, and we were often able to reduce the equilibration time to about 5 h, especially when we were using highly permeable Boise sandstone and a pressurized reservoir to flow through the sample at a very high rate.

**Fig. 3:**
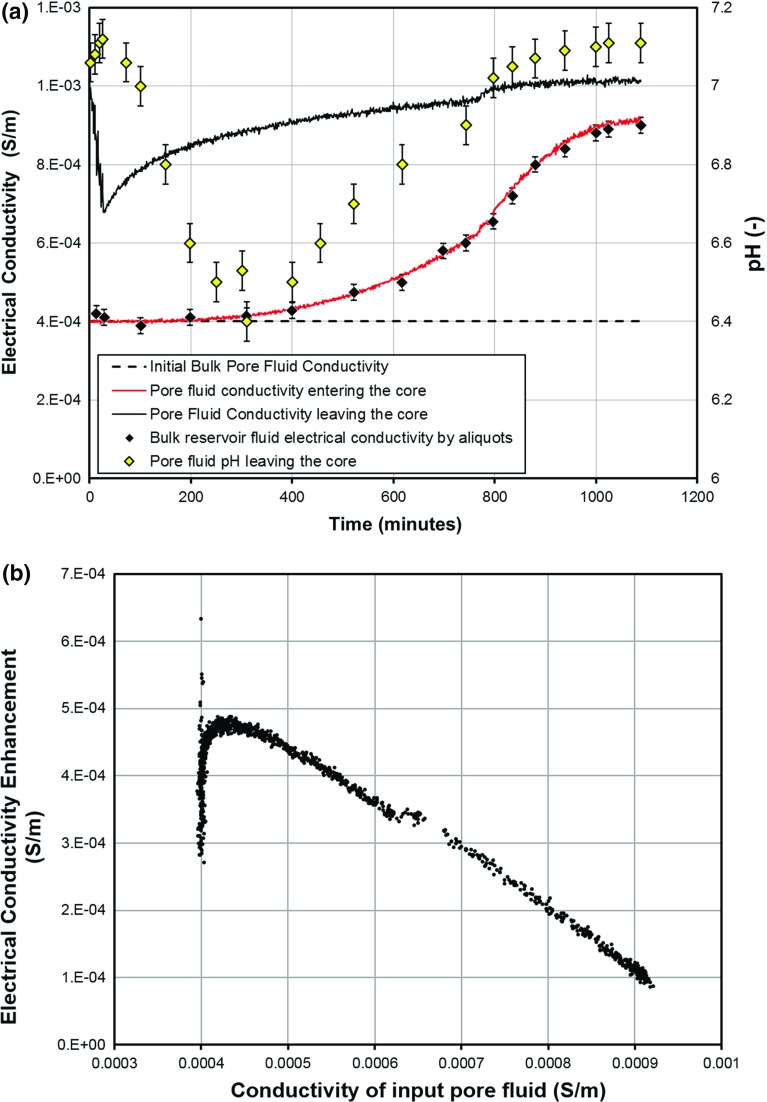
Example equilibration of electrical conductivity and pH for one of the measurement campaigns of sample BR2

Figure 3 shows the equilibration of a sample of Berea sandstone. The time at which equilibration is achieved is difficult to define. In our case we considered equilibration to have taken place when the difference between two consecutive running means over 10 measurements was less than the standard deviation in those same, but detrended, measurements. This is equivalent to noting that stability is judged when the systematic change is significantly less than the random errors in the measurements. This approach can be automated and avoids setting a threshold gradient below which equilibrium is achieved, which needs to be considered carefully and altered according to the magnitude of the measurements. In practice, we applied the method conservatively, often waiting for many more pore volumes to pass than was strictly necessary. The black line in Fig. 3a shows the conductivity of the pore fluid exiting the core, monitored every minute by an in-line device. Initially it is high as previously stagnant fluid is washed out of the sample, dropping towards the conductivity of the fluid in the large reservoir (dashed line), then increasing towards stability as the reservoir fluid comes into equilibrium with the rock by dissolution. The bump at 769 min was caused by a change in the thermal stability of the laboratory as winter heating was activated. The red line is the input conductivity, again monitored every minute by an in-line device. It starts at the reservoir (bulk) fluid conductivity and increases only gradually due to the reservoir’s large volume, and is relatively unaffected by the initial washout. The black diamonds are checks on this value made by taking aliquots of the reservoir fluid for measurement with a dipmeter. The difference between the conductivities of the input and output fluids is shown in Fig. 3b as a function of the conductivity of the input fluid and shows, as would be expected, that there is more dissolution when the input fluid is less saline, but also shows that the relationship between input fluid conductivity and the enhancement in the conductivity of that fluid by passing it through the rock is approximately linear. We conclude that equilibrium will be reached much faster if the input pore fluid has a conductivity and hence salinity which is close to that when equilibrium is achieved. This figure just confirms what we would expect by intuition. Figure 3 also shows the variation of pH during equilibration, which has a more complex and currently unexplained form.

The streaming potential and pressure difference measurements were taken during transient increases of the pressure difference and then during transient falls. Each of these measurements took about 100 s and logged over 10,000 individual measurements. Streaming potential measurements were taken with leak-free nonpolarizing reference Ag/AgCl electrodes (Warner Instruments 69-0053) and differentially amplified before being logged using a National Instruments DAQBoard NI USB6229 USB logger and PC running LabVIEW Signal Express. The streaming potential measurement system was calibrated using a Time Electronics 1017 Voltage/Current/Resistance Calibrator with a resolution of 10 nV, allowing us to make measurements to within an error of ± 1.2% when the streaming potential was over 1 mV. The measurement error dropped to ± 4.4% when the streaming potential was at the limit of its measurement at about 30 nV, which was caused by the signal dropping below the ambient noise and occurred for a salinity of about 4.5 mol/dm$^{3}$. The pressure measurements were taken using Omega PX302 transducers calibrated to an Endress and Hauser Deltabar S PMD75 secondary calibration standard and were also amplified by individual custom-designed amplifiers before being logged. The error in the differential measurement ranged from ± 3.4% at the lowest pressure differences measured to ± 1.2% at the highest. The electrical conductivity of the rock samples was made using the existing Ag/AgCl electrodes and a Solartron 1260A impedance spectrometer with the frequency fixed at 1 kHz.

Since the individual transient experiments were so easy and quick to carry out, we often carried out three or four in each direction (increasing and decreasing inlet pressure) in order to obtain very high-quality data. The fitting procedure also generates an individual fitting error, which we have combined with the measurement errors to obtain an overall experimental error for each measurement. The streaming potential data were plotted against the pressure difference measurements, all zeroes having been adjusted for. The streaming potential coefficient was then calculated from the gradient of the plot. This procedure was described in Walker et al. (2014), where such a plot can be found as their Fig. 2. The permeability was also measured when a steady pressure difference was attained at the end of the upward leg of the transient measurement. The electrical conductivity of the sample was measured immediately after at the end of the downward leg of the transient measurement after the vestiges of pressure difference had been removed using an equalizing valve.

## $C_\mathrm{sp}$ Results and Discussion

### Data Quality and Uncertainties

**Fig. 4:**
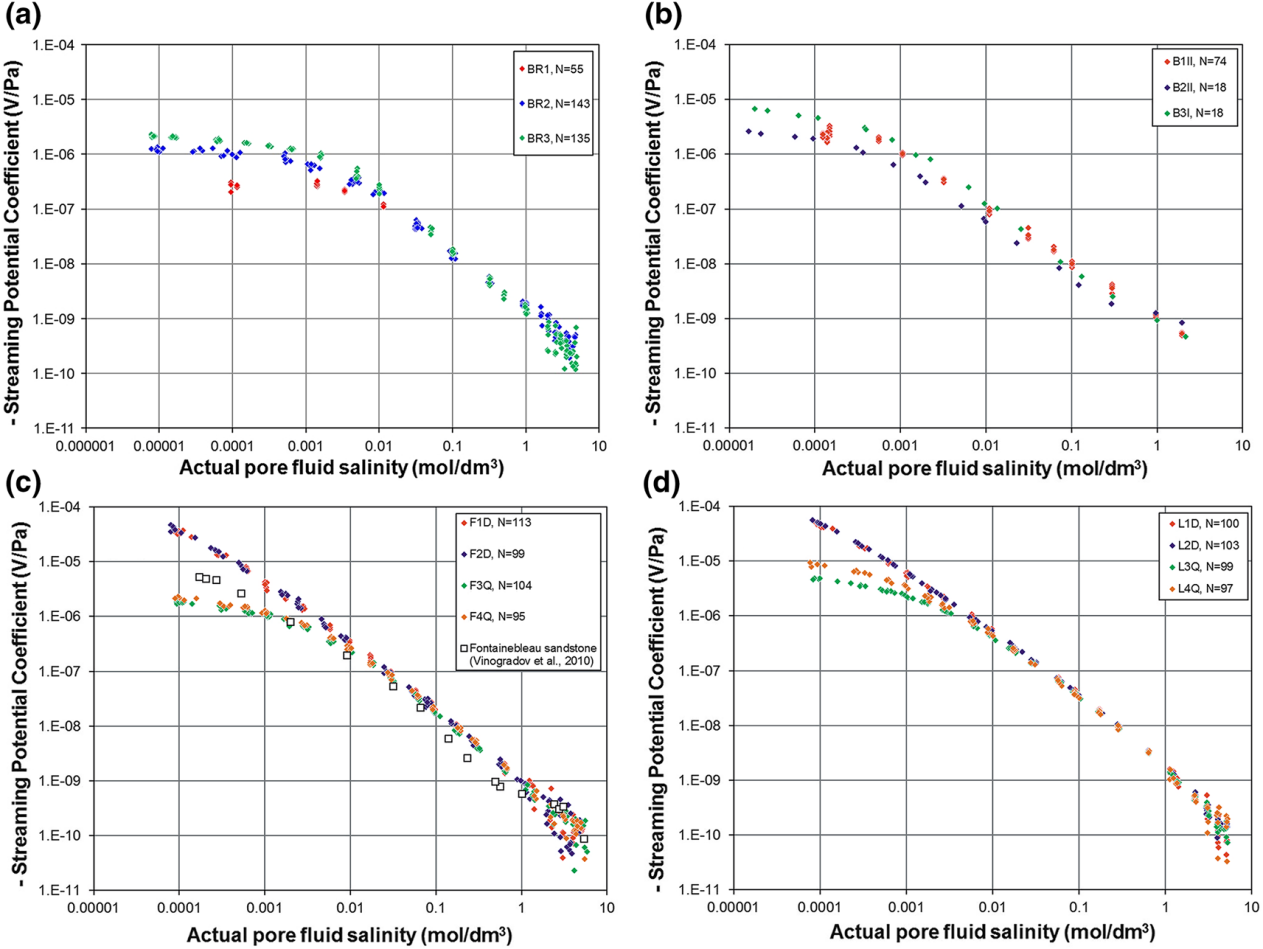
Measured streaming potential coefficients for samples of Berea, Boise, Fontainebleau, and Lochaline sandstones as a function of the salinity of the pore fluid in the rock at the time of the streaming potential measurement. **a** Berea sandstone (BR1, BR2, and BR3), **b** Boise sandstone (B1II, B2II, and B3I), **c** Fontainebleau sandstone (F1D, F2D, F3Q, and F4Q) together with the best previous data (Vinogradov et al. 2010) as open squares and **d** Lochaline sandstone (L1D, L2D, L3Q, and L4Q). In the cases of Fontainebleau and Lochaline sandstones red and blue symbols represent detrital form of the rock, while orange and green symbols represent the quartz overgrown type

**Fig. 5:**
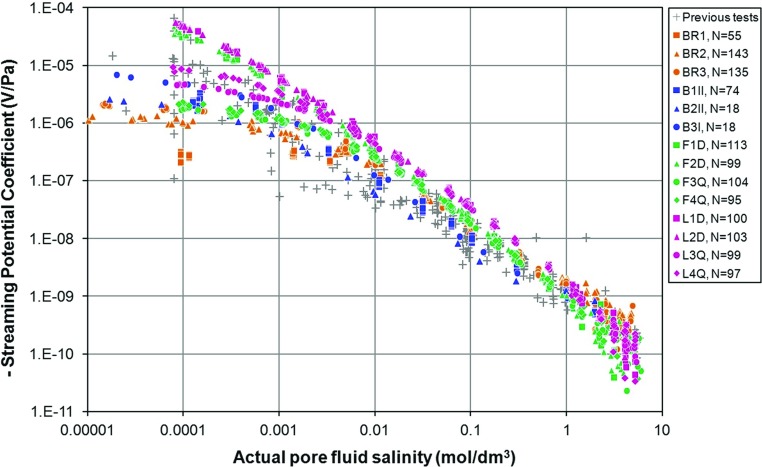
Aggregation of the new streaming potential coefficient measurements with those from the existing database. The existing data are shown undifferentiated by grey crosses, and the new data are shown as coloured symbols. Berea (red), Boise (blue), Fontainebleau (green), and Lochaline (purple)

Figure 4 shows the measured streaming potential coupling coefficients for all the samples as a function of the salinity of the fully equilibrated pore fluid that was in the sample at the time of the measurement. This figure contains 1253 individual measurements of streaming potential coefficient at a given fluid salinity, temperature and fluid pH, each of which is the result of about 10,000 determinations of streaming potential and fluid pressure difference. This data set represents a significant increase in the number of measurements available in the literature. The only previously available data of comparable quality to that generated in this work are for Fontainebleau sandstone by Vinogradov et al. (2010). These data have been included in Fig. 4c for comparison with the new determinations, showing that the two data sets are mutually consistent. Figure 5 shows a comparison of the new measurements with the database of 266 streaming potential coefficient measurements that were collated by Glover et al. (2012b) and which represent the majority of previously available measurements.

In general, it is clear that most of the new data are of relatively high quality with measurements for individual samples describing a smooth curve with little scatter. Berea sandstone BR1 and Boise sandstone B1II show lower-quality data that the other new data because they were made at the start of our measurement campaign and before our experimental protocols were fully developed. Nevertheless, the quality of these measurements also surpasses that of the previously existing data.

The uncertainties in all the measurements have been assessed and propagated where necessary. These uncertainties are not shown explicitly in Figs. 4 and 5 to avoid complicating already complex plots. However, all experimental errors are generally much smaller than most previous studies. At low and medium salinities the error in $C_{\mathrm{sp}}$ is approximately ± 5% of the measured value, while at high salinities, the error in $C_{\mathrm{sp}}$ is significantly higher, at about ± 45%. Nevertheless, the strong power law behaviour of $C_{\mathrm{sp}}$ ensures that its variation with salinity is clear. This observation is consistent with the scatter of each of the streaming potential trends.

The behaviour of the data can be split into three regimes: low salinity ($$C_{\mathrm{f}}<0.01\hbox { mol/dm}^{3})$$, medium salinity (0.01 mol/dm$$^{3}<C_{\mathrm{f}}<1$$ mol/dm$$^{3})$$, and high salinity ($$C_{\mathrm{f} }>1$$ mol/dm$$^{3})$$.

### Low Salinity Behaviour

At low salinities there is a flattening of the curves that has been ascribed to the microstructural properties of the rocks (Glover et al. 2012b). The flattening is more pronounced for the samples where there has been authigenic quartz overgrowth for both the Fontainebleau and Lochaline sandstones.

Comparison of the data with the supporting measurements shown in Table 1 shows that the flattening is more pronounced for samples with (i) lower porosity, (ii) larger cementation exponents, (iii) smaller grain sizes (and hence pore and pore throat sizes), and (iv) larger surface conduction.

This is consistent with the theoretical model of (Glover and Déry 2010; Glover et al. 2012b) and also implies that low salinity flattening of $C_{\mathrm{sp}}$ is more pronounced for rocks with larger formation factors *F* and electrical tortuosities $\tau _{\mathrm{e}}$ and hence for smaller connectedness *G* and connectivities $\chi$ (Glover 2010, 2017). The best example of this behaviour is the comparison of the results for the authigenic overgrowth forms of the Fontainebleau and Lochaline sandstones with respect to their chemically identical detrital forms. The four samples of overgrown sandstones are subject to a clear and pronounced flattening at high salinities compared to their analogous detrital samples. Reference to Table 1 shows that the authigenic quartz samples for both the Fontainebleau and Lochaline sandstones have (i) significantly lower porosities (a factor of 0.11 times lumping all porosity measurement types for both Fontainebleau and Lochaline samples), (ii) much higher formation factors (by a factor of 32.39), (iii) similar cementation exponents, (iv) similar grain sizes but much smaller pore throat sizes (by a factor of 0.062), and (v) much larger surface conduction (increased by a factor of 17.55). All of these differences are consistent with expectations from the theoretical model of Glover et al. (2012b).

Such functional dependencies would suggest that the sample measured by Vinogradov et al. (2010) and shown in Fig. 4c was predominantly detrital with incomplete development of authigenic quartz and a porosity and formation factor approximately midway between 2.4 and 22% and between 12 and 386, respectively. The values of these parameters reported by Vinogradov et al. (2010) were 7.2% and 157, respectively, which is consistent with our data and the expectations of the theoretical model.

It is worth noting that much of the scatter of the previously existing measurements at low salinities (shown by the grey crosses in Fig. 5) can be explained by invoking differing degrees of flattening that we now associate with the different porosity, cementation exponent, grain size, and specific surface conductivity of each sample. Since most of these parameters are not known because they either were not measured or reported, it is difficult to assess the degree to which the unknown microstructure of samples has led to the apparent large scatter of the previous streaming potential determinations. However, a sensitivity analysis of the Glover et al. (2012b) model should show the extent to which varying each parameter might lead to flattening in an attempt to be more confident in the assertion that this is the cause.

### Medium Salinity Behaviour

Our streaming potential coefficient data taken at medium salinities follow a smooth curve which approximates to a power law and manifests itself as a straight line with a negative gradient on bilogarithmic axes as shown in Figs. 4 and 5. There are slight indications that the streaming potential trends in this medium salinity range have additional structure, which has been noted in some samples before (Glover et al. 2012b).

**Fig. 6:**
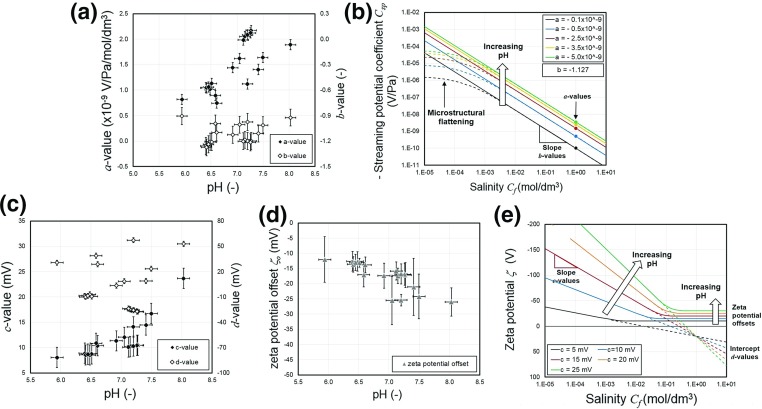
**a** The factor and exponent coefficients of the power law fit characterizing the medium salinity behaviour of streaming potential as a function of the pH of the pore fluid that was in equilibration with the rock sample. **b** Schematic diagram of the variation of $$C_\mathrm{sp}$$ with increasing pH. The value of *a* becomes larger, displacing the curves upwards, while the *d* remains approximately constant leading to no overall change of slope. **c** The factor and offset coefficients of the logarithmic law fit characterizing the low to medium salinity behaviour of zeta potential as a function of the pH of the pore fluid that was in equilibration with the rock sample. **d** The mean zeta potential offset calculated for all measurements with salinities greater than a visually defined threshold (Berea sandstone: 0.1 mol/dm$$^{3}$$, Boise sandstone: 0.01 mol/dm$$^{3}$$, Fontainebleau sandstone: 0.1 mol/dm$$^{3}$$, Lochaline sandstone: 1 mol/dm$$^{3})$$. Uncertainties represent the standard deviation of these data. **e **Schematic diagram of the variation of zeta potential with increasing pH. The value of *c* becomes larger, increasing the slope, the intercept *d* has a complex behaviour that can be positive or negative, and the zeta potential offset $$\zeta _\mathrm{o}$$ becomes more negative

We have carried out a simple power law fit to all of the new data in the salinity range 0.01 mol/dm$^{3}<C_{\mathrm{f}}<1$ mol/dm$^{3}$ and present the results in Table 2. The behaviour of these fits can be represented by the relationship

1 $$\begin{aligned} C_{\mathrm{sp}} =a C_\mathrm{f}^b , \end{aligned}$$

where the values of the coefficients *a* and *b* are given in Table 2 and the mean values for all our measurements are $\left\langle a \right\rangle = (-\,1.440\pm 0.517)\times 10^{-9}$ and $\left\langle b \right\rangle = -\,1.127\pm 0.125$. The large number of measurements in the data set, combined with their quality, ensures that the coefficients of determination for all individual power law fits are high, always exceeding 0.97. A fit to the same relationship carried out by Vinogradov et al. (2010) provided $a = -\,1.36\times 10 ^{-9}$ and $b = -\,0.9123$ with $R^{2} = 0.982$ with no uncertainties given and once their mV/MPa unit system has been converted into that used in this work (V/Pa). These values are consistent with our results. The extent to which such mean values are useful is, however, limited. One of the main controlling influences on streaming potential is zeta potential and that, as we will see in the next section, depends strongly on pH. Since the pore fluids in each of the measured samples has a distinct pH, it is perhaps unreasonable to read much into the mean values in Table 2 without knowing the pore fluid pH and its effect. Consequently, we have plotted the coefficients in Eq. (1) for individual samples as a function of the measured pore fluid pH in the first part of Fig. 6. This figure includes propagated experimental and fitting uncertainties for the coefficients and experimental uncertainties for the pH. It is clear that the factor coefficient *a* is sensitive to pH, varying between about 0.75 and about 2.2 over the pH range from 6 to 8, while the exponent coefficient *b* seems not to be sensitive to changes in pH in this range, taking a mean value of $-\,1.127\pm 0.125$. The consequence of these two dependencies is that increasing the pH tends to displace the streaming potential curves in Figs. 4 and 5 upwards without appreciably altering their slope (when plotted on bilogarithmic axes) as shown schematically in Fig. 6b.

**Table 2:** Medium salinity behaviour of streaming potential and zeta potential

Sample code	Rock type	*pH*	*a *($\times ~10^{-9}$ )	*b*	${{R}}^{2}$	*c*	*d*	${R}^{2}$	Zeta offset
			Equation (1)			Equation (3)			(mV)
BR1	Berea sandstone	8.030	1.890	$-$ 0.923	0.9891	23.66	52.88	0.8127	$-$ 26.00
BR2		7.050	1.620	$-$ 1.008	0.9916	12.04	8.64	0.9239	$-$ 25.52
BR3		7.410	1.406	$-$ 1.110	0.9946	14.45	8.89	0.9723	$-$ 21.05
Mean		7.497	1.639	$-$ 1.014	$-$	16.72	23.47	$-$	$-$ 24.19
SD		0.496	0.243	0.093	$-$	6.133	25.47	$-$	2.73
B1II	Boise sandstone	7.200	1.120	$-$ 0.9757	0.9945	14.1	57.3	0.9085	$-$ 25.40
B2II		5.940	0.818	$-$ 0.9059	0.9745	8.05	30.59	0.9795	$-$ 12.03
B3I		6.610	0.743	$-$ 1.101	0.9899	10.37	28.76	0.9958	$-$ 13.79
Mean		6.583	0.894	$-$ 0.994	$-$	10.84	38.88	$-$	$-$ 17.07
SD		0.630	0.200	0.099	$-$	3.052	15.98	$-$	7.26
F1D	Fontainebleau sandstone	6.400	1.047	$-$ 1.259	0.9966	8.88	$-$ 8.76	0.9882	$-$ 12.64
F2D		6.410	1.045	$-$ 1.275	0.9997	8.54	$-$ 9.97	0.9881	$-$ 13.64
F3Q		6.480	1.014	$-$ 1.207	0.9981	8.53	$-$ 6.87	0.9928	$-$ 12.64
F4Q		6.510	1.131	$-$ 1.212	0.9984	8.76	$-$ 9.36	0.9962	$-$ 13.10
Mean		6.450	1.059	$-$ 1.238	$-$	8.678	$-$ 8.74	$-$	$-$13.01
SD		0.054	0.050	0.034	$-$	0.172	1.341	$-$	0.48
L1D	Lochaline sandstone	7.270	2.171	$-$ 1.200	0.9986	10.46	$-$ 27.51	0.9887	$-$ 17.13
L2D		7.260	2.122	$-$ 1.215	0.9989	10.44	$-$ 27.32	0.9935	$-$ 16.89
L3Q		7.150	2.053	$-$ 1.186	0.9810	10.19	$-$ 24.48	0.9943	$-$ 17.39
L4Q		7.120	1.984	$-$ 1.202	0.9986	10.09	$-$ 23.72	0.9779	$-$ 15.84
Mean		7.200	2.083	$-$ 1.201	$-$	10.29	$-$ 25.76	$-$	$-$ 16.81
SD		0.076	0.082	0.012	$-$	0.184	1.940	$-$	0.68
Mean	Overall behaviour	6.917	1.440	$-$ 1.127	$-$	11.33	3.505	$-$	$-$ 17.36
SD		0.544	0.517	0.125	$-$	4.060	28.823	$-$	5.11

*SD* standard deviation

## High Salinity Behaviour

The measurements for salinities greater than 1 mol/dm$^{3}$ for all samples are in good agreement with the measurements of Jaafar et al. (2009) and Vinogradov et al. (2010) up to 5.55 mol/dm$^{3}$ (the maximum salinity at which measurements were taken in this work). The behaviour is characterized by a continuation of the power law relationships that exists at medium salinities, which is manifested in bilogarithmic plots as a continuing reducing linear trend and in linear space as a tendency towards zero as the salinity approaches full saturation. This behaviour ensures that the streaming potential does not change polarities at high salinities for the range of pHs studied in this work.

It should be noted that the measurements of streaming potential in the high salinity regime (Figs. 4, 5) have a much higher uncertainty than at lower salinities. This is due to the extreme difficulty in making such measurements, particularly in keeping the pore fluid stable, as noted by Jaafar et al. (2009) and Vinogradov et al. (2010).

## Zeta Potentials

### Calculation Methodologies

Zeta potentials have been calculated from each streaming potential coefficient measurement using the Helmholtz–Smoluchowski law (Glover 2015a) with Overbeek’s correction (Jouniaux and Pozzi 1995; Walker et al. 2014), together with experimentally measured supporting data and empirical models. This calculation requires the knowledge of the electrical conductivity ($\sigma _{\mathrm{f}})$, permittivity ($\varepsilon _{\mathrm{f}})$, and viscosity $({\eta }_{\mathrm{f}})$ of the equilibrated pore fluid as well as the formation factor of the rock both at high salinities ($F_{\mathrm{o}})$ and at each of the salinities used in the measurement ($F_{\mathrm{i}})$. Since the effective conductivity of the saturated sample was also measured independently both at the experimental salinity $({\sigma }_{\mathrm{i}})$ and at high salinity $({\sigma }_{\mathrm{eff}})$, the formation factor at each experimental salinity ($F_\mathrm{i} ={\sigma }_{\mathrm{f}}/{\sigma }_{\mathrm{i}})$ and that at high salinity $(F_{\mathrm{o}}={\sigma }_{\mathrm{f}}/{\sigma }_{\mathrm{eff}})$ could be calculated. The ratio of the formation factor at high salinity (i.e. when the contribution of surface conduction is negligible) to that at the given salinity of the experiments (i.e. $F_{\mathrm{o}}/F_{\mathrm{i}})$ is termed the Overbeek’s correction. The full equation for calculating the zeta potential including the Overbeek correction is given by

2 $$\begin{aligned} \zeta =\frac{C_{\mathrm{sp}} \eta _\mathrm{f} \sigma _\mathrm{f} }{\varepsilon _\mathrm{f}}\frac{F_\mathrm{o} }{F_\mathrm{i} }. \end{aligned}$$

Other parameters used in the calculation were electrical permittivity, which was calculated for an aqueous NaCl solution for each salinity at $25^{\,\circ }\hbox {C}$ using the unpublished method of Gary Olhoeft (e.g. Glover et al. 2012b) and pore fluid viscosity, which was calculated for a NaCl solution for each salinity at $25^{\,\circ }\hbox {C}$ using the method of Phillips et al. (1978).

## Data Quality and Uncertainties

**Fig. 7:**
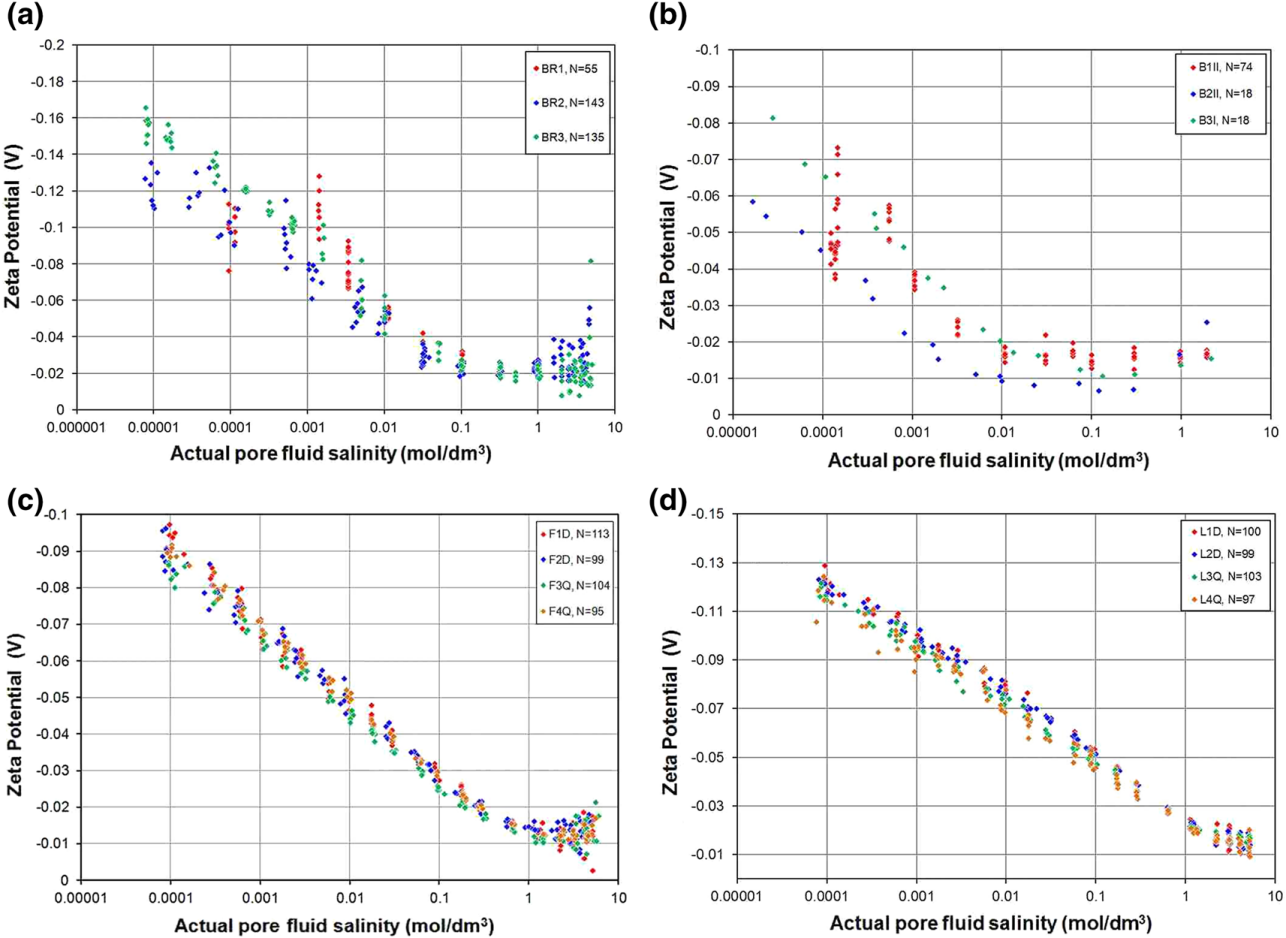
Derived zeta potentials for samples of Berea, Boise, Fontainebleau, and Lochaline sandstones as a function of the salinity of the pore fluid in the rock at the time of the streaming potential measurement. **a** Berea sandstone (BR1, BR2, and BR3), **b** Boise sandstone (B1II, B2II, and B3I), **c** Fontainebleau sandstone (F1D, F2D, F3Q, and F4Q), and **d** Lochaline sandstone (L1D, L2D, L3Q, and L4Q). For Fontainebleau and Lochaline sandstones solid symbols represent the detrital form of the rock, while open symbols represent the quartz overgrown type

**Fig. 8:**
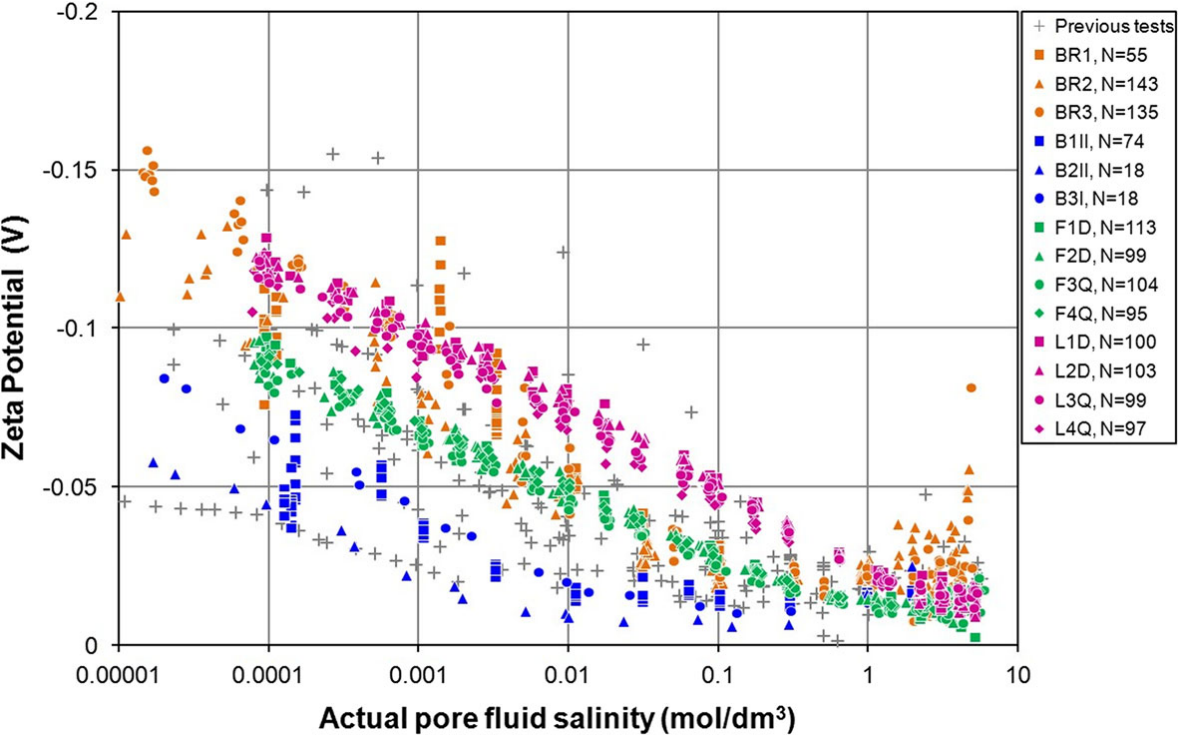
Aggregation of the new derived zeta potential measurements with those from the existing database. The existing data are shown undifferentiated by grey crosses; the new data are shown as coloured symbols. Berea (red), Boise (blue), Fontainebleau (green), and Lochaline (purple)

Figure 7 shows the resulting zeta potential as a function of the salinity of the fully equilibrated pore fluid for all the samples. These measurements are compared with the database of 269 derived zeta potential values that were collated from the literature by Glover et al. (2012b) in Fig. 8.

Figure 7 shows that the new zeta potential data for each of the rock types form trends, where trends were often difficult to make out in the previously published data due to inaccurate measurements or the lack of control of key parameters. The knowledge and control of pH was a particular problem in these previously published data, as exemplified in Fig. 1b. We ascribe the relatively small scatter in our data to the control we have imposed on the pH of the solutions we used. For example, the main reason for the contrast between the values of zeta potential for the Fontainebleau sandstone (green symbols) and Lochaline sandstone (purple symbols) in Fig. 8 arises from a difference in the pH of the solutions with which each was saturated. These solutions had a pH of $6.45\pm 0.05$ and $7.2 \pm 0.08$ ($\hbox {mean}\pm \hbox {range}$), respectively (Tables 1, 2). In fact, modelling shows that it is the pH that is the main control on the zeta potential (e.g. Hunter 1981; Glover et al. 2012b). We hypothesize that the scatter in all of the existing measurements (shown by the grey crosses in Fig. 8), can be explained by suggesting that these measurements were taken at a variable but unknown pore fluid pH somewhere between 5 and 9. The papers from which these previous data were gathered often mention that the fluid pH was within this range but rarely specify the measured value, remaining sparse, ambiguous and approximate with their pH information (Fig. 1b).

Unfortunately, the sparsity of pH data in the previous publications ensures that we cannot test the hypothesis that it is the pH that causes the scatter in the previous data because the fluid pH is either ill-controlled or not disclosed. We can, however, test the hypothesis by examining our new data, where the pH is well-controlled, and comparing it with theoretical models (Glover and Déry 2010; Glover et al. 2012b). We consider the variation of zeta potential with pH later in this paper, but unfortunately there is insufficient space to include full modelling of the new data in this paper. However, such modelling will be reported in a forthcoming paper. A simple example of the control that pH has on the zeta potential is given by comparing the curves for our Boise B2II and Boise B3I samples (Fig. 5b). The mean and standard deviations for the pH of the equilibrated fluids for these two samples were $5.94\pm 0.24$ and $6.61 \pm 0.086$, respectively, leading to a clear separation in the zeta potential curves.

It is worth noting that, unlike the $C_{\mathrm{sp}}$, the zeta potential does not depend on rock microstructure at low salinities. This implies that the cause of the low salinity flattening in the streaming potential coefficient for some rock samples is to be found in the way the rock microstructure affects surface conductance rather than being associated with changes in the surface conductivity itself.

The consequence of the lack of a different zeta potential behaviour at low salinities is that the zeta potential can be split into two regimes, one which describes the behaviour in the low to medium salinity range, and the other which is valid for high salinities.

### Low to Medium Salinity Behaviour

For the low to medium salinities the zeta potential decreases linearly with the logarithm of salinity. We have carried out a simple logarithmic fit to all of the new data in the salinity range for which Fig. 7 shows this behaviour to occur. This was $C_{\mathrm{f}}<0.1$ mol/dm$^{3}$ for Berea sandstone, $C_{\mathrm{f}}<0.01$ mol/dm$^{3}$ for Boise sandstone, and $C_{\mathrm{f}}<1$ mol/dm$^{3}$ for the Fontainebleau and Lochaline sandstones. The mean behaviour of these fits can be represented by the relationship

3 $$\begin{aligned} \zeta = c \log _{10} \left( {C_\mathrm{f} } \right) + d, \end{aligned}$$

where the values of the coefficients *c* and *d* are given in Table 2 and the mean values for all our measurements are $\left\langle c \right\rangle = 11.33 \pm 4.06$ mV and $\left\langle d \right\rangle = 3.505 \pm 28.823$ mV. Once again the large number of measurements in the data set combined with their quality ensures that the coefficients of determination for all individual power law fits are high, usually exceeding 0.97, but dropping below that value for two samples where the data were not as high quality.

A number of authors have fitted Eq. (3) or its formal equivalent to their data. Vinogradov et al. (2010) obtained $c = 19.02$ mV, $d = -\,9.67$ mV, Pride and Morgan (1991) obtained $c = 26$ mV and $d = -\,8$ mV, Bolève et al. (2007) suggested $c = 29.1$ and $d = -\,14.6$ mV, while Revil et al. (1999) have estimated the values at $c \approx 20$ mV and $d \approx -\,10$ mV. Jaafar et al. (2009), which shares some of its data with Vinogradov et al. (2010) suggested $c = 20.85$ mV and $d =-\,6.43$ mV. In all of these works, the uncertainty on the values was not calculated and often the values obtained are for an aggregation of different samples and rock types at uncontrolled pH. Nevertheless, all values of *c* occur in the 10–30 mV range and all values of *d* occur in the $-\,5$ to $-\,15$ mV range and are not obviously consistent with the values we obtain, which we ascribe to a pH dependence.

While it is clear that zeta potential depends strongly on pH from Figs. 7 and 8 and Table 2 alone, we wished to examine how the zeta potential depended upon pH in the pH 6–8 range. Consequently, we have plotted the coefficients in Eq. (3) against the measured pH in the third part of Fig. 6. This figure includes propagated experimental and fitting uncertainties in the same way as the analogous diagram for streaming potential that was described previously. It is clear from Fig. 6c that the coefficient *c* varies in a well-defined manner that all samples follow and which is extremely sensitive to pH, varying nonlinearly between about 8.05 mV and about 23.66 mV over the pH range from 6 to 8 and covering the range of values obtained by other authors. By contrast the offset coefficient *d* shows a more scattered behaviour, with different rock types producing different trends, and both positive and negative values. There is some indication of a weak dependence on pH for the Berea and the Boise sandstones, with positive values, while the Fontainebleau and Lochaline samples provide mean values of *d* that are consistent with the values from other authors.

The consequence of these two dependencies is that increasing the pH tends to increase the slope of the low to medium salinity zeta potential curves in Figs. 7 and 8, while producing both positive and negative values for the intercept as shown in the schematic diagram in Fig. 6e.

### High Salinity Behaviour

Continuation of the trend described in the last section to high salinities would result in the zeta potential changing polarity (from negative to positive). However, this has not been observed in any of the experimental determinations made in this work or in that of Jaafar et al. (2009) and Vinogradov et al. (2010). Instead, at high salinities the zeta potential appears to level off at a small constant negative value or even increase slightly (i.e. become more negative). Previously such behaviour had not been noted by Pride and Morgan (1991), Bolève et al. (2007) or Revil et al. (1999) because they had not considered high salinities and had consequently not observed the levelling-off behaviour.

The cause of the asymptotic behaviour of the zeta potential has been discussed by Jaafar et al. (2009) and Vinogradov et al. (2010). They noted that the start of the constant zeta potential behaviour occurs at about 0.4 mol/$\mathrm{dm}^\mathrm{3}$, at which salinity the Debye length is about 0.47 nm and approximates to the size of a hydrated sodium ion. This observation leads them to suggest that the constant zeta potential at high salinities reflects the maximum charge density in the diffuse layer which is reached when the diffuse layer thickness approaches the diameter of the counterions. In this model at high salinities the double layer resembles the Helmholtz model, where there is a single layer of hydrated, immobile ions in the Stern layer, and a single layer of hydrated, mobile ions composing the diffuse layer (i.e. a parallel plate capacitor model). They noted that this hypothesis is supported by their data, with the limiting value of the zeta potential occurring at about $-\,20$ mV (Jaafar et al. 2009) and $-\,17$ mV (Vinogradov et al. 2010). Previous measurements made by Dukhin et al. (2005) on $\alpha$-alumina provided a value of about $-\,10$ mV, while those of Johnson et al. (1999a, b), also for $\alpha$-alumina, gave values between $-\,20$ and $-\,30$ mV at 1 mol/dm$^{3}$. In this work we have obtained values of the high salinity zeta potential which are $-\,24.19\pm 2.73$ mV for Berea sandstone, $-\,17.07\pm 7.26$ mV for Boise sandstone, $-\,13.01\pm 0.48$ mV for Fontainebleau sandstone, and $-\,16.81\pm 0.68$ mV for Lochaline sandstone. The mean value for all of our high salinity measurements taken together is $-\,17.36 \pm 5.11$ mV, all of which agree well with measurements made by other researchers.

Supporting information for the existence of this constant value of zeta potential at high salinities is provided from modelling studies. It has been observed that the electrochemical model developed by Glover and Déry (2010), Glover et al. (2012b), and Glover (2015b) cannot provide good fits to zeta potential and streaming potential data unless a so-called zeta potential offset is introduced, which is formally the same as the zeta potential at high salinities. The parameter is necessary because the equations for zeta potential (Revil and Glover 1997, 1998; Revil et al. 1999) cannot model the Helmholtz behaviour. When included in modelling Glover et al. 2012b found that zeta potential offsets between $-\,10$ and $-\,35$ mV allowed model curves to fit the experimental data, with values depending on the rock type, while Glover (2015b) found a value of $-\,15$ mV fitted the data of Luong and Sprik (2014) well.

Nevertheless, the data in all of our tests and previous tests were made on rocks that were predominantly silica and with an aqueous NaCl solution. It would be expected that they would provide similar zeta potential offsets. Consequently, we hypothesized that pH could also have a role in determining the zeta potential offset (i.e. the value of the zeta potential at high salinity). We have plotted the zeta potential offset against the pH that was measured in this work on fully equilibrated pore fluid. The mean value of zeta potential offset for each sample is shown in Fig. 6d with uncertainties based on the standard deviation of the measurements made at high salinities. This figure suggests a slight but clear increase in the magnitude of the zeta potential offset with increasing pH, from about $-\,5$ mV to about $-\,35$ mV. Consequently, it is possible to explain the entire range of high salinity zeta potential offset behaviour observed by all the authors who have observed it by invoking changes in the pore fluid pH, and the pH-dependent behaviour of the zeta potential offset is consistent with the pH dependence found in the medium to low salinity behaviour discussed previously. The zeta potential offset behaviour has been incorporated in the schematic diagram showing the effect of increasing pH on the zeta potential curves (Fig. 6e).

## Conclusions

High-quality streaming potential measurements have been taken on fourteen samples of four types of sandstone (Berea, Boise, Fontainebleau, and Lochaline) as a function of pore fluid salinity from 10$^{-5}$ to 4.5 mol/dm$^{3}$, amounting to over 1253 individual measurements in total. This database easily surpasses the total number of previous measurements on silica-based rocks and provides an effective set of data for the testing of theoretical models, which will be the subject of a forthcoming paper. Measurements were taken on sandstones with a number of different compositions and pore structures, represented by detrital sandstones and sandstones with authigenic quartz overgrowths in both Fontainebleau and Lochaline sandstones.

The 313 measurements of streaming potential coefficient that were taken at salinities greater than 1 mol/dm$^{3}$ (an 8.83-fold increase in available data) confirm the behaviour observed by Jaafar et al. (2009) and Vinogradov et al. (2010), and are consistent with their interpretation of the development of a maximum charge density at high salinities. The low salinity behaviour of streaming potential coefficient shows a sensitivity to the microstructural properties of the rock (porosity, grain size, formation factor), which is particularly clear in the samples with authigenic quartz overgrowths. The streaming potential coefficient is sensitive to the pore fluid pH.

Zeta potential was calculated from each of the streaming potential coefficient measurements and showed a variation that was highly sensitive to both salinity and pH. The database of 269 existing measurements had been widely spread and difficult to interpret, and though it was suspected that pore fluid pH was a factor, the lack of pH control, measurement, and discussion in previous experimental papers meant that it was impossible to quantify the effect of pH. This data set shows the pH dependence clearly and suggests that the constant high salinity zeta potential (zeta potential offset) also depends on pH.

While the data presented in this work represent, we believe, a significant step forward in the availability of data which can be used to test the developing models, much remains to be done experimentally. There is the need for experiments using divalent brines and at raised temperatures. In addition, clay minerals are also thought to be important in controlling many salinity-dependent processes in sandstones, such as formation damage and controlled salinity water injection. Consequently, more experimental data examining the effect of clays on the streaming potential coefficients and zeta potentials of clastic rocks. Finally, few measurements are available in carbonates, and these experiments are complicated by rock–fluid interaction needs to be carried out. In summary, there is scope for much more experimental work, all of which will have important implications for water, hydrocarbon, and waste disposal industries.

## Electronic supplementary material

Below is the link to the electronic supplementary material.

Supplementary material 1 (xlsx 286 KB)

## References

[CR1] Alkafeef SF, Alajmi AF (2007). The electrical conductivity and surface conduction of consolidated rock cores. J. Colloid Interface Sci..

[CR2] Avena MJ, de Pauli CP (1998). Proton adsorption and electrokinetics of an Argentinean montmorillonite. J. Colloid Interface Sci..

[CR3] Block GI, Harris JG (2006). Conductivity dependence of seismoelectric wave phenomena in fluid-saturated sediments. J. Geophys. Res..

[CR4] Bolève A, Crespy A, Revil A, Janod F, Mattiuzzo JL (2007). Streaming potentials of granular media: influence of the Dukhin and Reynolds numbers. J. Geophys. Res..

[CR5] Churcher, P.L., French, P.R., Shaw, J.C., Schramm, L.L.: Rock properties of Berea sandstone, Baker dolomite, and Indiana limestone. Soc. Pet. Eng. (1991). 10.2118/21044-MS

[CR6] Dukhin A, Dukhin S, Goetz P (2005). Electrokinetics at high ionic strength and hypothesis of the double layer with zero surface charge. Langmuir.

[CR7] Gaudin AM, Fuerstenau DW (1955). Streaming potential studies: quartz flotation with anionic collectors. Trans. Am. Inst. Min. Metall. Eng..

[CR8] Glover PWJ (2010). A generalized Archie’s law for n phases. Geophysics.

[CR9] Glover PWJ (2015). Electrical Properties. Treatise on Geophysics.

[CR10] Glover, P.W.J.: Comment on “examination of a theoretical model of streaming potential coupling coefficient”. Int. J. Geophys. (2015b). 10.1155/2015/941246

[CR11] Glover, P.W.J.: A new theoretical interpretation of Archie’s saturation exponent. Solid Earth Discuss. (2017). 10.5194/se-2017-5

[CR12] Glover PWJ, Déry N (2010). Dependence of streaming potential on grain diameter and pore radius for quartz glass beads. Geophysics.

[CR13] Glover PWJ, Walker E (2009). A grain size to effective pore size transformation derived from an electro-kinetic theory. Geophysics.

[CR14] Glover PWJ, Zadjali II, Frew KA (2006). Permeability prediction from MICP and NMR data using an electrokinetic approach. Geophysics.

[CR15] Glover, P.W.J., Walker, E., Ruel, J., Tardif, E.: Frequency-dependent streaming potential of porous media—part 2: experimental measurement of unconsolidated materials. Int. J. Geophys. (2012a). 10.1155/2012/728495

[CR16] Glover PWJ, Walker E, Jackson MD (2012). Streaming-potential coefficient of reservoir rock: a theoretical model. Geophysics.

[CR17] Gomez C, Dvorkin J, Vanorio T (2010). Laboratory measurements of porosity, permeability, resistivity and velocity on Fontainebleau sandstones. Geophysics.

[CR18] Guichet X, Jouniaux L, Pozzi JP (2003). Streaming potential of a sand column in partial saturation conditions. J. Geophys. Res..

[CR19] Hunter RJ (1981). Zeta Potential in Colloid Science.

[CR20] Jaafar MZ, Vinogradov J, Jackson MD (2009). Measurement of streaming potential coupling coefficient in sandstones saturated with high salinity NaCl brine. Geophys. Res. Lett..

[CR21] Jiang YG, Shan FK, Jin HM, Zhou LW (1998). A method for measuring electro-kinetic coefficients of porous media and its potential application in hydrocarbon exploration. Geophys. Res. Lett..

[CR22] Johnson SB, Scales PJ, Healy TW (1999). The binding of monovalent electrolyte ions on $$\alpha $$-alumina: I. Electroacoustic studies at high electrolyte concentrations. Langmuir.

[CR23] Johnson SB, Franks GV, Scales PJ, Healy TW (1999). The binding of monovalent electrolyte ions on $$\alpha $$-alumina: II. The shear yield stress of concentrated suspensions. Langmuir.

[CR24] Jouniaux L, Pozzi JP (1995). Streaming potential and permeability of saturated sandstones under triaxial stress: consequences for electrotelluric anomalies prior to earthquakes. J. Geophys. Res..

[CR25] Jouniaux L, Pozzi JP (1995). Permeability dependence of streaming potential in rocks for various fluid conductivities. Geophys. Res. Lett..

[CR26] Jouniaux L, Pozzi JP (1997). Laboratory measurements anomalous 0.1–0.5 Hz streaming potential under geochemical changes: implications for electrotelluric precursors to earthquakes. J. Geophys. Res..

[CR27] Kirby BJ, Hasselbrink EF (2004). Zeta potential of microfluidic substrates. 1. Theory, experimental techniques, and effects on separations. Electrophoresis.

[CR28] Kosmulski M, Dahlsten P (2006). High ionic strength electrokinetics of clay minerals. Colloids Surf. A Physicochem. Eng. Asp..

[CR29] Kosmulski M, Mączka E, Janusz W, Rosenholm JB (2002). Multi-instrument study of the electrophoretic mobility of quartz. J. Colloid Interface Sci..

[CR30] Li HC, de Bruyn PL (1966). Electrokinetic and adsorption studies on quartz. Surf. Sci..

[CR31] Li SX, Pengra DB, Wong PZ (1995). Onsager’s reciprocal relation and the hydraulic permeability of porous media. Phys. Rev. E.

[CR32] Lorne B, Perrier F, Avouac J-P (1999). Streaming potential measurements: 1. Properties of the electrical double layer from crushed rock samples. J. Geophys. Res..

[CR33] Luong, D.T., Sprik, R.: Examination of a theoretical model of streaming potential coupling coefficient. Int. J. Geophys. (2014). 10.1155/2014/471819

[CR34] Morgan FD, Williams ER, Madden TR (1989). Streaming potential properties of Westerly Granite with applications. J. Geophys. Res..

[CR35] Pasqualini D, Heitmann K, TenCate JA, Habib S, Higdon D, Johnson PA (2007). Nonequilibrium and nonlinear dynamics in Berea and Fontainebleau sandstones: low-strain regime. J. Geophys. Res..

[CR36] Pengra DB, Li SX, Wong PZ (1999). Determination of rock properties by low-frequency AC electrokinetics. J. Geophys. Res..

[CR37] Phillips, S.L., Ozbek, H., Otto, R.J.: Basic energy properties of electrolytic solutions database. In: Sixth International CODATA Conference Santa Flavia (Palermo), Sicily, Italy, May 22–25. http://www.osti.gov/bridge/purl.cover.jsp;jsessionid=3954E775156A8BC0FA35DB5CE5B402D4?purl=/6269880-iPJPhB/ (1978). Accessed 10 June 2010

[CR38] Poirier, J.E., Cases, J.M.: Sur l’origine et la nature de l’interaction adsorbat-adsorbant dans les systèmes à interactions faibles. Solid–liquid interactions in porous media; Technip Editions 447–462 (1985)

[CR39] Pride SR, Morgan FD (1991). Electrokinetic dissipation induced by seismic waves. Geophysics.

[CR40] Revil A, Glover PWJ (1997). Theory of ionic surface electrical conduction in porous media. Phys. Rev. B.

[CR41] Revil A, Glover PWJ (1998). Nature of surface electrical conductivity in natural sands, sandstones, and clays. Geophys. Res. Lett..

[CR42] Revil A, Pezard PA, Glover PWJ (1999). Streaming potential in porous media. I. Theory of the zeta-potential. J. Geophys. Res..

[CR43] Revil A, Hermitte D, Spangenberg E, Cochemé JJ (2002). Electrical properties of zeolitized volcaniclastic materials. J. Geophys. Res..

[CR44] Revil A, Saracco G, Labazuy P (2003). The volcano-electric effect. J. Geophys. Res..

[CR45] Sen P, Goode P (1992). Influence of temperature on electrical conductivity on shaly sands. Geophysics.

[CR46] Sen P, Goode P (1992). Erratam to: Influence of temperature on electrical conductivity of shaly sands. Geophysics.

[CR47] Sprunt ES, Mercer TB, Djabbarah NF (1994). Streaming potential from multiphase flow. Geophysics.

[CR48] Tardif E, Glover PWJ, Ruel J (2011). Frequency-dependent streaming potential of Ottawa sand. J. Geophys. Res. B.

[CR49] Tosha T, Matsushima N, Ishido T (2003). Zeta potential measured for an intact granite sample at temperatures to $$200^{\circ }{\rm C}$$. Geophys. Res. Lett..

[CR50] van der Heyden FHJ, Stein D, Besteman K, Lemay SG, Dekker C (2006). Charge inversion at high ionic strength studied by streaming currents. Phys. Rev. Lett..

[CR51] Vinogradov J, Jaafar MZ, Jackson MD (2010). Measurement of streaming potential coupling coefficient in sandstones saturated with natural and artificial brines at high salinity. J. Geophys. Res..

[CR52] Walker, E., Glover, P.W.J., Ruel, J.: A transient method for measuring the DC streaming potential coefficient of porous and fractured rocks. J. Geophys. Res. (2014). 10.1002/2013JB010579

[CR53] Will G, Nover G (1986). Measurement of the frequency dependence of the electrical conductivity and some other petro-physical parameters of core samples from the Konzen (West Germany) drill hole:. Ann. Geophys..

[CR54] Worden R, Morad S (2009). Quartz Cementation in Sandstones, Special Publication 29 of the IAS.

